# Influence of Single-Wall Carbon Nanotube Suspension on the Mechanical Properties of Polymeric Films and Electrospun Scaffolds

**DOI:** 10.3390/ijms241311092

**Published:** 2023-07-04

**Authors:** Anna A. Dokuchaeva, Sergey V. Vladimirov, Vsevolod P. Borodin, Elena V. Karpova, Andrey A. Vaver, Gleb E. Shiliaev, Dmitry S. Chebochakov, Vasily A. Kuznetsov, Nikolay V. Surovtsev, Sergey V. Adichtchev, Alexander G. Malikov, Mikhail A. Gulov, Irina Y. Zhuravleva

**Affiliations:** 1 Institute of Experimental Biology and Medicine, Federal State Budgetary Institution National Medical Research Center Named after Academician E.N. Meshalkin of the Ministry of Health of the Russian Federation, 15 Rechkunovskaya St., Novosibirsk 630055, Russia; a_dokuchaeva@meshalkin.ru (A.A.D.); vladimirov_s@meshalkin.ru (S.V.V.); borodin_v@meshalkin.ru (V.P.B.); vaver_a@meshalkin.ru (A.A.V.); 2 Group of Optical Spectrometry, Center of Spectral Investigations, N.N. Vorozhtsov Novosibirsk Institute of Organic Chemistry SB RAS, 9 Lavrentiev Avenue, Novosibirsk 630090, Russia; karpovae@nioch.nsc.ru; 3 LLC “Tuball Center NSK”, 24 Inzhenernaya St., Novosibirsk 630090, Russia; shilyaev.ge@swcnt.net (G.E.S.); dmitry.chebochakov@swcnt.net (D.S.C.); 4 I.Ya. Postovsky Insititute of Organic Synthesis of the Ural Branch of the Russian Academy of Sciences (IOS UB RAS), S. Kovalevskoy St., 22/20, Yekaterinburg 620108, Russia; basilkv@yandex.ru; 5 Institute of Automation and Electrometry of the Siberian Branch of the Russian Academy of Sciences, Academician Koptyug Avenue, 1, Novosibirsk 630090, Russia; snv@iae.nsk.su (N.V.S.); adish@ngs.ru (S.V.A.); 6 Khristianovich Institute of Theoretical and Applied Mechanics of the Siberian Branch of the Russian Academy of Sciences, Institutskaya Str. 4/1, Novosibirsk 630090, Russia; smalik@ngs.ru (A.G.M.); gulovy@mail.ru (M.A.G.)

**Keywords:** carbon nanotubes, PCL, PCHC, tubular scaffolds, bioengineering

## Abstract

Carbon nanotubes (CNTs) are used in applications ranging from electrical engineering to medical device manufacturing. It is well known that the addition of nanotubes can influence the mechanical properties of various industrial materials, including plastics. Electrospinning is a popular method for fabricating nanomaterials, widely suggested for polymer scaffold manufacturing. In this study, we aimed to describe the influence of single-walled carbon nanotube (SWCNT) suspensions on polymeric poured films and electrospun scaffolds and to investigate their structural and mechanical properties obtained from various compositions. To obtain films and electrospun scaffolds of 8 mm diameter, we used poly-ε-caprolactone (PCL) and poly(cyclohexene carbonate) (PCHC) solutions containing several mass fractions of SWCNT. The samples were characterized using tensile tests, atomic force and scanning electronic microscopy (AFM and SEM). All the studied SWCNT concentrations were shown to decrease the extensibility and strength of electrospun scaffolds, so SWCNT use was considered unsuitable for this technique. The 0.01% mass fraction of SWCNT in PCL films increased the polymer strength, while fractions of 0.03% and more significantly decreased the polymer strength and extensibility compared to the undoped polymer. The PHCH polymeric films showed a similar behavior with an extremum at 0.02% concentration for strength at break.

## 1. Introduction

Biodegradable polymer scaffolds have become a popular concept in tissue engineering [1,2,3]. This approach to the fabrication of artificial tissue combines the benefits of easy processing, financial accessibility, variability of materials and incredible potential for substrate and process modification [3,4,5]. However, an ideal polymeric vascular scaffold has not yet been created. Despite the abundance of research in this field, most artificial vessel prototypes have serious limitations, such as unsuitable mechanical properties, poor vascular compliance, slow biodegradation rate, low cytocompatibility and others [3,6,7]. Mechanical behavior is one of the most common problems of polymeric vascular scaffolds. The optimal scaffold should fully resemble a native vessel and remain sustainable throughout the implantation period without thrombosis and interruptions, and at the same time, it should be a temporary support matrix for cell ingrowth that will degrade later on.

PCL is a biodegradable polymer that is well known in bioengineering. It was offered for vascular tissue engineering in 1986 [8] and remains an object of high scientific interest today [9,10,11]. PCL degrades to non-toxic 6-hydroxyhexanoic acid via hydrolysis in vivo within 2 to 3 years [12]. Tubular PCL grafts have demonstrated good endothelialization and moderate foreign body reaction in both short- and long-term experiments in rat models [13,14]. For a long time, PCL scaffolds were considered one of the best options for vascular replacement, but they still did not repeat the native vessel response to blood flow, as they were too stiff [14,15]. In search of better mechanical properties, several PCLs and their processing modifications were realized: polymeric chain elongation, use of various solvents [16,17,18], working solution additives [19], preparation of soluble mixtures with other polymeric components [20] and alternative processing [21]. These changes have significantly affected the properties of the grafts, but they can still be improved.

PCHC is an amorphous conductive biodegradable CO_2_-based polycarbonate [22] known for its non-toxic degradation products [23]. It has been proposed as a vector for gene and drug delivery [24,25] and tissue scaffold manufacturing [26]. Although it is a perspective material, pure PCHC is rarely used in tissue engineering because of its low glass transition temperature, high stiffness, low tensile strength, slow biodegradation and poor cell adhesion [27], but many copolymers with semi-crystalline plastics such as vinyl, PCL and poly(lactic acid) (PLA) were made on its basis [28,29,30].

Electrospinning is a common technique suitable for processing carbon nanotubes, polymeric solutions and their mixtures [31,32]. The great advantage of this method is the ability to regulate the fiber diameter and pore size of the non-woven matrix by means of simple adjustments to the setup parameters [33]. Structures reproducing the architecture of the natural extracellular matrix (ECM) can be obtained in this way [34]. At the same time, many materials and their composites with desirable properties can be extremely difficult to process or, due to their specific features, such as solution viscosity, low conductivity or glass transition temperature, cannot be subjected to electrospinning [35,36,37].

Many of these problems could probably be solved through creating nanocomposite materials with nanotubes [38]. Nanotubes are another advanced topic of contemporary medicine [39,40]. They are being tested as possible substrates for drug delivery [41,42] and antibacterial [43], hemophobic [44] or hemostatic coatings [45]. This highly adjustable material can vary its behavior depending on morphology, weight and size [46]. This may be the key to the development of a new complex material that combines the resilient properties of nanotubes and the biocompatibility of well-tested polymers.

The addition of CNTs is a simple way to regulate the rheological properties of polymer solutions, although the effects greatly depend on the CNT concentration, their size and dispersion, and also on the polymer structure. The most obvious effect of CNTs concerns their conductive properties. Being mixed with non-conducting polymer, dispersed CNTs form a percolated network that increases the conductivity of the substrate. At the same time, the dispersion of nanotubes in polymeric solutions depends on the polymer molecular weight and macro- and microstructure determining the polymer/nanofiller interactions, which is the reason for specific effects in certain polymer/nanofiller combinations [47]. The electrical properties of CNTs also lead to their alignment within the scaffold during electrospinning, unlike their spreading in polymer films, which builds a different kind of network [48,49].

It is also noteworthy that multiwall carbon nanotubes (MWCNTs) are more widespread among biomedical research groups than single-wall nanotubes (SWCNTs). To date, the number of publications devoted to MWCNTs significantly exceeds the number of papers devoted to SWCNTs. Perhaps this can be explained by the complexity and lower accessibility of SWCNTs [50].

In this research, we aimed to investigate the effect of SWCNT suspension on the nanofiber morphology and mechanical properties of PCL and PCHC vascular scaffolds and poured films.

## 2. Results and Discussion

**Figure 1:**
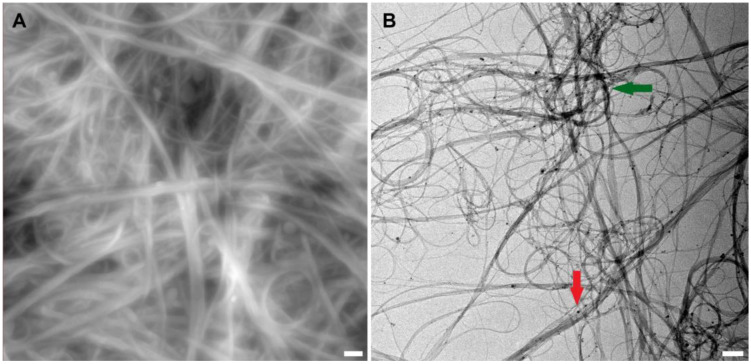
Atomic force microscopy (AFM) image of nanotube bundles in suspension (**A**) and transmission electron microscopy (TEM) image of plain nanotubes (the image is courtesy of LLC “Plasma-Chemical Technologies”) (**B**). Scale bars are 200 nm. The green arrow indicates the point of SWCNT clustering; the red arrow indicates the thin SWCNT bundles agglomerating into a larger one.

To obtain images of nanotube bundles using atomic force microscopy, a drop of the suspension was placed on a clean glass plate and dried. An example of an atomic force microscopy image is shown in Figure 1A.

One of the problems researchers face when preparing nanotubes is their propensity to agglomerate or cluster. This phenomenon is explained by the strong molecular attraction between the nanotubes. Van der Waals forces cause entanglement, which leads to CNTs bundling together [51]. The long hair-like structures of different thicknesses seen in the images (Figure 1) are the result of this process. A forming cluster and SWCNT bundles, agglomerating into a thicker filament, are presented in Figure 1B.

Figure 1A demonstrates the SWCNT bundles on the surface of a dried drop of the studied suspension. The bundles in this microphotograph are even larger because of the impact of the dried CNT dispersant additive—HNBR. It is an uncured rubber polymer with a linear molecular structure, highly tropic to the nanotube surface. This affinity to CNTs may be explained by anchoring CN groups and double bonds in their structure. The role of this component is to enclose separated CNTs and their bundles, keeping them dispersed through preventing reverse adhesion in suspension. However, when dried, it causes additional clumping due to its own physical properties. The HNBR coating cannot be seen on the surface of the bundles at this magnification because of its subtlety.

**Figure 2:**
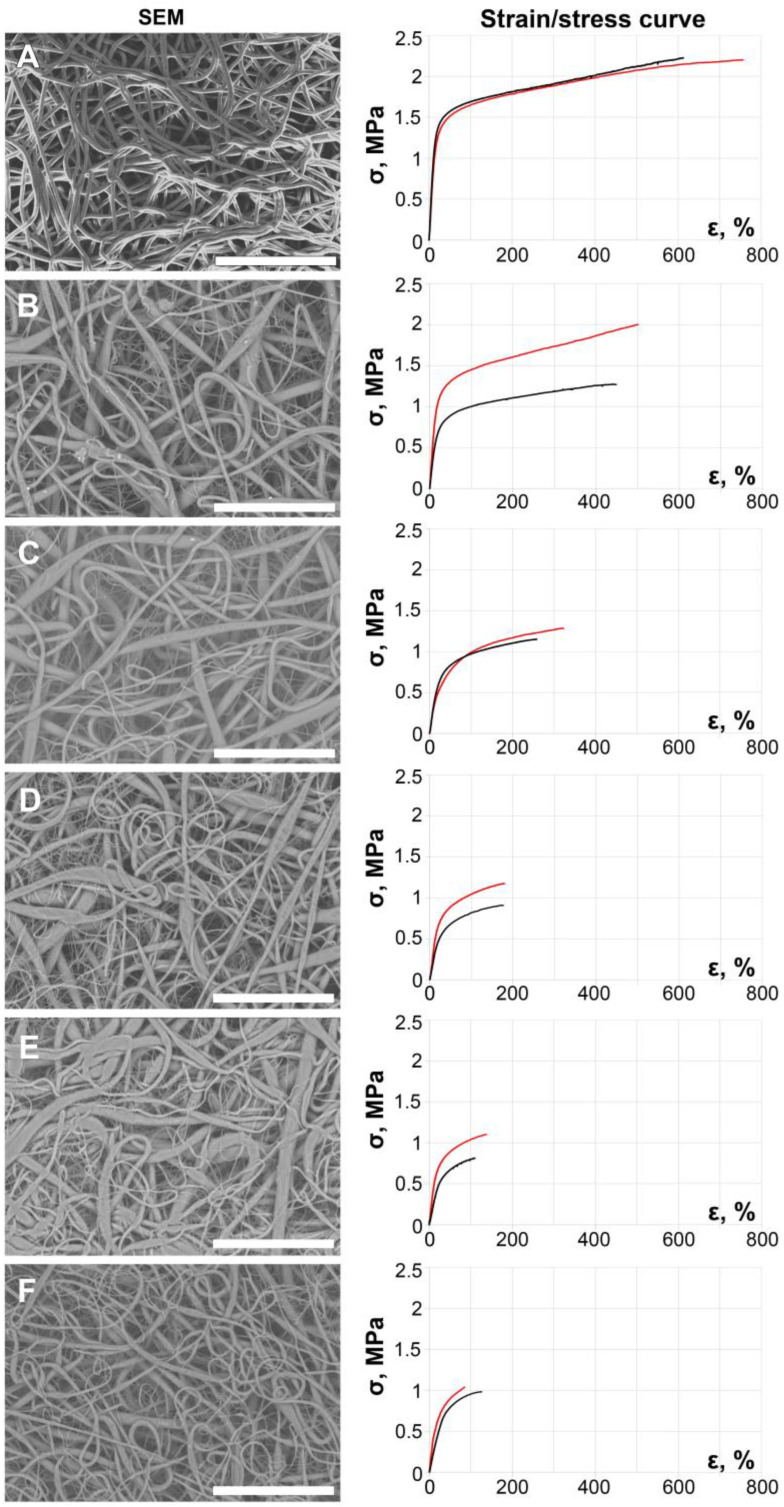
SEM images and corresponding typical strain-stress curves of obtained electrospun PCL scaffolds: plain 10% PCL solution in pure chloroform (**A**) and working solutions containing 0.01 (**B**), 0.02 (**C**), 0.03 (**D**), 0.04 (**E**) and 0.05% (**F**) CNT by weight. Scale bar is 100 µm. The red lines represent the stress–strain stretching in the axial direction; black curves correspond to circumferential stretching.

All the obtained PCL scaffolds demonstrated nanofibers organized in a dense looped pattern without any alignment (Figure 2). It is clearly seen that with the SWCNT additives (Figure 2B–F), electrospun fibers become diversified by thickness. The diameters of pure PCL fibers ranged from 1.03 to 5.71 µm, but the difference between the maximum and minimum fiber diameter in the group containing only 0.01% of nanotubes by weight of dry polymer varied from 1.33 to 10.4 µm. The largest difference was found in samples with 0.05% SWCNTs: the thinnest fibers were 177 nm in diameter, which is comparable to the diameter of large CNT bundles, while the largest ones were 10.3 µm. A wide range of fiber diameters within the groups demonstrates the featured fiber irregularity, increasing proportionally with SWCNT content.

The surface of fiber-to-collector contacts increases with increasing SWCNT concentration from 0.01% to 0.04% by weight. However, in scaffolds containing 0.05% of the nanotubes, the surface of fiber-to-collector contacts decreases, but still does not reach the structure of pure PCL.

We assume that the increasing concentration of SWCNTs in working solutions increases the number of fibers emitted from one polymer jet and fiber-to-fiber connections, which are mostly represented by a thicker “main” fiber interlaced with thin “additional” ones. The number of “additional” fibers increases with the rise in the SWCNT content while their thickness changes inversely proportional to it. This effect might be due to the nanotubes’ high conductivity: we suggested that, as the main polymer jet emits forming a larger thread, the thin, more conductive bundles of nanotubes separate from it, moving with speed higher than the main flow. At the same time, the scaffolds containing the nanotube suspension demonstrate featured irregularity of the “main” filaments.

The addition of a SWCNT suspension complicates the electrospinning process: solutions with nanotubes are prone to dripping, instability of the Taylor cone and fiber interruption; it also takes more time for the fabricated scaffolds to dry. Although these solutions are viscous enough to be electrosopun, their behavior is similar to that of PCL solutions with lower molecular mass [52]. The supporting properties of these fibers seem to deteriorate in solutions of 0.01–0.04%, corresponding with a rise in CNT concentration; the result of this feature in combination with slow drying is fiber melting, which can be seen on the SEM images (Figure 2). The solution with 0.05% mass fraction of SWCNTs formed a stable Taylor cone and was easier to process, but filament interruption still took place.

**Figure 3:**
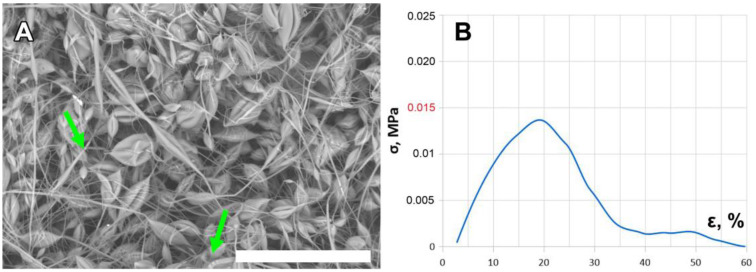
SEM image of the obtained electrospun PCHC scaffold (**A**) and the corresponding axial strain–stress curve (**B**). Green arrows indicate fiber disruptions. Red color indicates the low SBR value for these matrices (below 15 kPa). Scale bar is 100 µm.

PCHC solutions can be electrospun (Figure 3) [26], despite the unfavorable physical properties described above, but we have not been able to obtain a sustainable tubular electrospun scaffold with or without SWCNTs. All these scaffolds were too fragile to be taken off the collector without damaging the structure. Tensile tests also could not be carried out effectively due to their fragility.

Figure 3A represents the microstructure of the electrospun PCHC scaffold. The obtained matrix is built of bead-on-string fibers of various diameters and lengths with numerous fiber interruptions. Most sites of filament breakage have a specific shape of stacked discs (Figure 3A), which implies that the fiber was damaged while drying. Increasing the PCHC weight fraction up to 20% and adding a nanotube suspension did not improve the scaffold properties. This result amply demonstrates the brittleness of PCHC nanofibers. Although there are reports of successful PCHC scaffold fabrication [26], we found that the physical properties of the tested polymer (atactic PCHC, Mn = 25 kDa) are not appropriate for the fabrication of tubular scaffolds. This statement was confirmed by the strain–stress curve for stretching in the axial direction (Figure 3B). The tensile strength at break (SBR) value for these matrices was as low as nearly 15 kPa, hardly comparable to that of the PCL samples (Figure 2).

**Figure 4:**
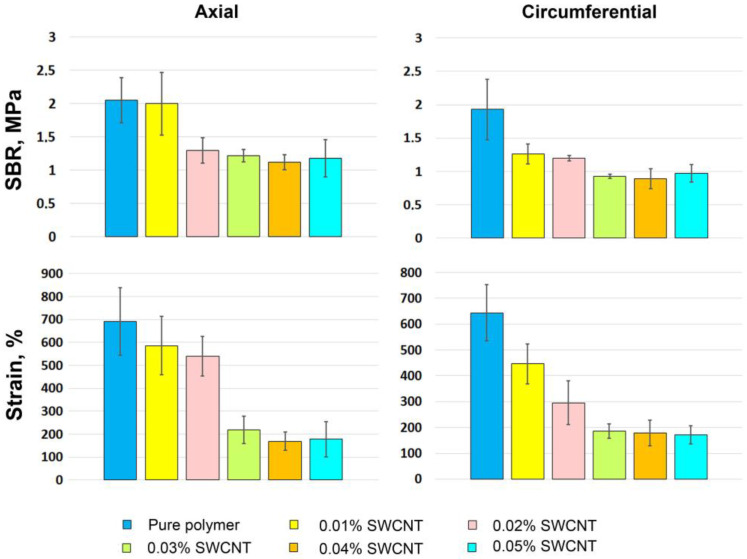
Mechanical properties of PCL electrospun tubular scaffolds. The data are presented as mean ± standard deviation.

The mean tensile strength at break (SBR) and strain values for each group of PCL electrospun scaffolds are presented in Figure 4; *p*-values are given in Tables S1–S4. The samples were tested in the axial and circumferential directions relative to the central axis of the tubular scaffolds.

These data (Figure 4) and stress–strain curves (Figure 2 and Figure S1, Tables S1–S4) describing mechanical behavior of the tested samples prove the negative effect of the SWCNT suspension on electrospun PCL scaffolds. The impact of 0.01% of SWCNTs on PCL fibers was not statistically proven (Tables S1–S4). The effect of 0.02% of SWCNTs on axially oriented PCL fibers was manifested in a significant decrease in SBR (*p* = 0.008) and their extensibility in circumferential direction (*p* = 0.034). Further, these indicators decreased as the concentration of nanotubes increased, but the extensibility decreased more gradually than SBR in the SWCNT concentration range of 0.01–0.03%. For PCL with 0.03–0.05% nanotube additives, the SBR and strain were almost the same (*p* > 0.05). When tested in axial direction, the mean SBR was significantly lower only with the 0.02% SWCNT concentration compared to the pure polymer (*p* = 0.008) and the 0.01% group (*p* = 0.025). The axial extensibility of the control, 0.01% and 0.02% groups varied in the range of 550–700 MPa and did not differ significantly. Thus, with the rise in the SWCNT content, the tendency of scaffolds being less strong and extensible enhances, the difference in values between the last three groups is not significant, but all of them show poor results when compared to first three groups (*p*-values are given in Tables S1–S4). Though, such behavior may be explained by the nanoarchitecture of these scaffolds, in particular, by the number and the thickness of “additional” filaments and non-melted main threads. The loss of scaffold strength from pure PCL to 0.05% nanotubes suspension is likely due to the amount of aligned SWCNTs inside the threads, since ordered nanotube bundles build a more fragile structure. The same pattern can be seen on the strain–stress curves given in Figure 2, which correlates with the degree of fiber irregularity seen in the SEM images.

We suggest that the nanotubes alignment occurred during the electrospinning of the material. The reason for this may be the high conductivity of SWCNTs and their ordering under the application of the electrostatic field [31].

**Figure 5:**
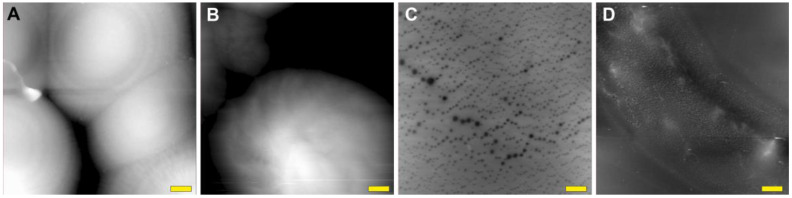
Atomic force microscopy images of the obtained films: pure PCL (**A**), PCL + 0.05% SWCNT (**B**), pure PCHC (**C**) and PCHC + 0.05% SWCNT (**D**). Scale bars are 10 µm.

Figure 5 demonstrates the surfaces of the obtained films, each of which has its own specific relief. Pure PCL films consist of connected round ring-banded formations building linear constrictions in spherulite impingement areas. The surface of these globules is covered with small ridges arranged in concentric circles. PCL films containing 0.05% of SWCNTs is also represented by circular shapes, smaller in diameter and connected in the same way. The surface of these formations is covered with spirally oriented pile-like protrusions, which are more prominent than on pure polymer surface. We suggest that this is the result of enhanced spherulite lamellae twist. Similar results were found by another research group, which relates this phenomenon to the CNTs location inside the spherulite. During the crystal formation, chains of PCL molecule arrange into lamellae, but CNTs are not involved in this process, gathering between the lamellae or their bundles. Consequently, more unbalanced surface stresses of lamellae or bundles, induces the twisting of lamellae [53].

Unlike PCL, PCHC films have a flat structure molted with small pores of various diameter. With addition of SWCNTs, the size of the pores decreased, but no other changes in the film structure were noticed. This may be due to the SWCNT network formation in a layer of fully amorphous atactic PCHC polymer media. We must note that stereoregular poly(cyclohexene carbonate)s have a different crystallization behavior [54].

**Figure 6:**
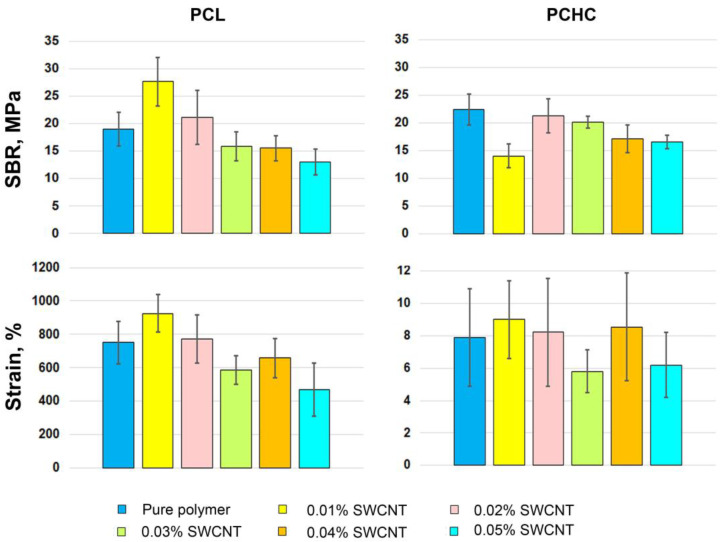
Mechanical properties of obtained PCL and PCHC films. The data are presented as mean ± standard deviation.

Polymeric films demonstrate different mechanical behavior than electrospun scaffolds (Figure 6, Figures S2 and S3). For PCL film samples, the addition of 0.01% of SWCNTs led to the best SBR result compared with all the other samples including the control (*p* < 0.05, Table S5). The mean strain in this group was also higher than in the control sample, but no statistical difference was found between them (*p* = 0.22, Table S6).

The mechanical properties of the 0.02% SWCNT group were similar to those of pure PCL; no significant difference was found between them. Resembling the trend, noted for tubular scaffold analysis, three last concentrations of nanotube suspension decreased the film tensile strength and elongation capacity when compared to pure polymer, 0.01 and 0.02% CNT concentrations, while samples of the 0.05% group were the least durable and extensible. The tendency of smaller amounts of SWCNT having a positive effect on the polymers’ mechanical properties was proven by other research groups, the main difference of opinions on this subject is the amount of CNT chosen as optimal [38,55], what may depend on the different choices of a polymer.

The evaluation of PCHC films was certainly difficult because of the physical features described above, such as low strength and high fragility. The results collected for this polymer were also controversial. The group containing 0.01% of SWCNTs demonstrated a significant decrease in SBR among all of the other samples (*p* = 0.0001 when compared to pure polymer, *p*-values are represented in Table S6). The SBR of the 0.02% group was almost equal to the control, but with a smooth trend to SBR decreasing at higher concentration. The strain values were highly variable within groups and mean values are not differ significantly (Table S8).

Probably the vast difference in mechanical behavior depends on the distribution of the SWCNTs. In films, they lie along the initial polymer structure rather than in a line, as it was in electrospun samples. Deliberate orientation of nanotubes in a film gives the polymer-SWCNT compound a “reserve of flexibility” because the applied tensile force is distributed on the network of differently oriented bundles, and its vector is directed at various angles relating to them. This feature and specificity of macromolecular polymeric structures define the CNT network pattern and needed CNT concentration.

Since the electrospinning method is considered unsuitable for processing these solutions, because of the major decrease in SBR and extension capacity (Figure 4), the alternative ways of matrix formation should be sought. One of the most common methods not involving conductive features of nanotubes or chemical additives is molding. This method makes it possible to create scaffolds of complex shape, which cannot be obtained using electrospinning. Along with this, it has a minimal effect on the orientation of nanotubes and the structure of the polymer. The main limitations of molding with polymeric solutions are related to the requirements for molds: they should be resistant to the used solvents, chemically inert, and easily removable.

It is important to mention that in this research, the results cannot refer solely to the SWCNT concentration. As we noted earlier [47], the uniform dispersion of nanotubes in the working solution is crucial for nanofilling/polymer reactions and, therefore, the overall effect on the raw material. This suggested our use of a stabilizing agent, HNBR, can influence the mechanical properties of the obtained samples, and at the same time its use is highly substantial.

Through adding the SWCNT suspension in a concentration of 0.01% CNT/dry polymer, we also introduce the solution to higher concentrations of HNBR, which needs a more “holistic” approach in research. It also opens new space for suspension modification; the search for another stabilizing agent can lead to significant findings in this field.

**Figure 7:**
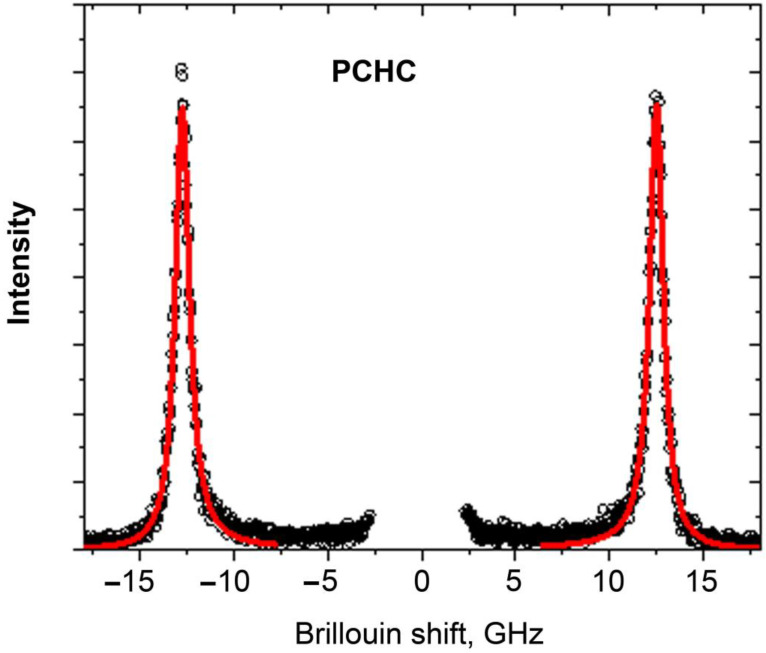
Representative Brillouin spectrum of the PCHC sample (circles). Lines are DHO fits.

Figure 7 shows a representative Brillouin spectrum of the PCHC sample. The Stokes and anti-Stokes parts of the spectrum show the peaks corresponding to inelastic scattering involving acoustic phonons.

The position of the maximum, ***ν****_B_*, and the linewidth Γ*_B_* of the Brillouin peaks were determined from the experimental spectra *I(**ν**)* using the damped harmonic oscillator (DHO) function:

$$I\left(\nu \right)=\frac{I_{0}}{\pi }\frac{\Gamma_{B}\nu_{B}^{2}}{{\left(\nu^{2}-\nu_{B}^{2}\right)}^{2}+{\left(\Gamma_{B}\nu \right)}^{2}}$$

This fit was applied for the Brillouin spectra of the PCHC and PCL series with various concentrations of SWCNTs. Results of this analysis for ***ν****_B_* are presented in Table 1.

It can be seen that both polymers demonstrate an increase in the position of the Brillouin peak with increasing SWCNT concentration. This increase is weak and about 0.8% for PCL and 3% for PCHC for the whole range of SWCNT concentrations (from 0 to 0.05%).

For the backscattering geometry, the Brillouin peak maximum ***ν****_B_* determines the longitudinal elastic modulus *M_B_* as

$$M_{B}=\rho {\left(\frac{\lambda \nu_{B}}{2n}\right)}^{2}$$

where *n* is the refractive index, *ρ* is the material density and *λ* is the laser wavelength. Neglecting the effects for *n* and *ρ* at low SWCNT concentrations (from 0 to 0.05%), the addition of SWCNTs provides a relative change of *M_B_* as

$$M_{B}/M_{B}\;\left(0\%\right)=\frac{\nu_{B}^{2}}{\nu_{B}^{2}\left(0\%\right)}\;\;\;$$

**Figure 8:**
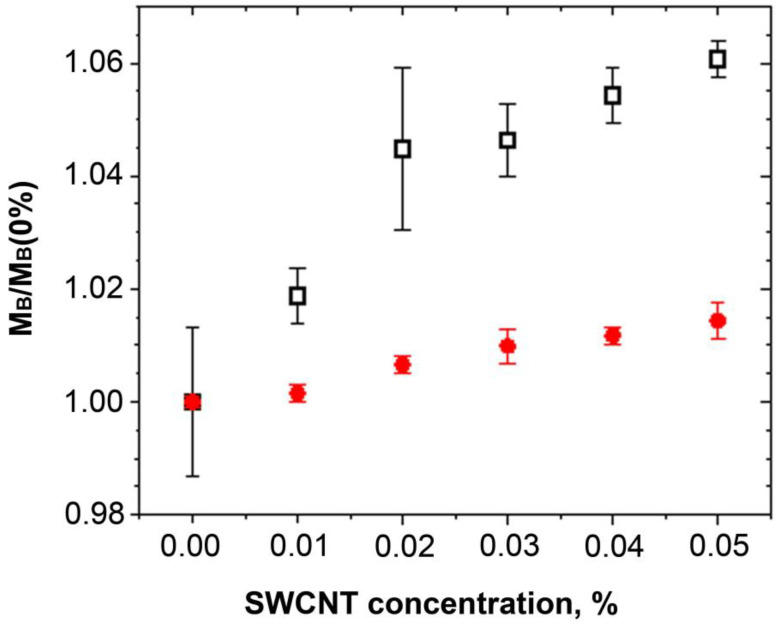
The relative change of M_B_ versus SWCNT concentrations for PCHC (squares) and PCL (circles) polymers. The data are presented as mean ± standard deviation.

The relative change of *M_B_* versus SWCNT concentration is shown in Figure 8.

It is seen that there is an increase in the infinite-frequency elastic modulus with SWCNT concentration. These results allow us to conclude that in Figure 6, the trend towards a decrease in the low-frequency Young’s modulus with increasing SWCNT concentration above 0.02% is caused by the changes in the relaxation response.

**Figure 9:**
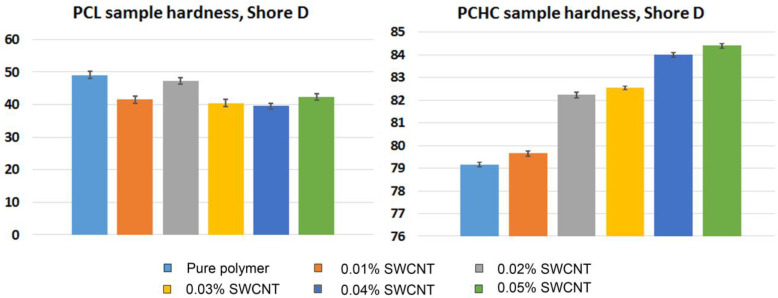
Visualization of durometer Shore D hardness data of tested samples. The data are presented as mean ± standard deviation.

The durometer Shore D hardness data of tested samples are presented in Figure 9. For the PCL samples, all of the probes demonstrate a decrease in durometer Shore D hardness values, compared to the original polymer. Most of these specimens have durometer Shore D hardness in the range of 40–42 except for the group with the 0.02% SWCNT concentration, which has higher values than the rest of CNT-containing samples, much closer to the original polymer (49 in the control group and 47.25 for the 0.02% SWCNT group). This behavior also suggests that for PCL samples, there is an optimum CNT content, which can improve the mechanical properties of this polymer, while addition of excessive amounts of the suspension can have negative effects. Another case is the PCHC samples, for which durometer Shore D hardness increases proportionally to the suspension concentration.

Among these samples, the lowest durometer Shore D hardness belongs to pure PCHC (79.15). With rise in CNT concentration, it gradually increases up to 84.4 at the concentration of 0.05%. In terms of electrospinning process this effect can result in higher solution conductivity, but also in an increase in fiber brittleness.

## 3. Materials and Methods

### 3.1. Nanotube Suspension Preparation

Single-walled carbon nanotubes (SWCNTs) with an average diameter of 1.6 nm obtained from LLC “Plasma-Chemical Technologies” (Novosibirsk, Russia) were evaluated via optical absorption spectroscopy on a Shimadzu UV-3600 spectrophotometer (Shimadzu Corporation, Kyoto, Japan) according to ISO 10868:2017 [56]. To achieve the fine dispersion of SWCNTs, a suspension in 1-methoxy-2-propyl acetate (Jiangsu Sanmu Group Co., Ltd., Yixing, China) with hydrogenated nitrile butadiene rubber (HNBR) Therban 2004 (Arlanxeo, Pittsburg, PA, USA) as dispersing and stabilizing agent was prepared using the ultrasonication technique. The SWCNT content in the obtained suspension was 0.1 wt.%; HNBR content was 0.5 wt.%.

### 3.2. Polymer Composition

For the PCL working solution mixture, 1 g of 3 mm spherical pellets of (1.7)-polyoxepan-2-one (ε-polycaprolactone) with a molecular mass (Mn) of 80 kDa (cat. No 440744, Sigma-Aldrich Co., St. Louis, MO, USA) was collected in a probe tube, and then a desired amount of nanotube suspension was added: in the test groups, the weight ratios of nanotubes to dry PCL were 0.01, 0.02, 0.03, 0.04 and 0.05%; control samples were made of pure PCL. After that, pure chloroform (Vekton, Saint Petersburg, Russia) was added to obtain the 10% PCL solution by weight. Tubes with probes were kept at 37 °C with permanent shaking until the polymer was completely dissolved. Each portion was prepared separately on the date of scaffold fabrication. The PCHC working solution was made in a similar way using 1 g of poly(cyclohexene carbonate) with a molecular mass (Mn) of 25 kDa unsized pellets (Empowered materials, New Castle, DE, USA).

### 3.3. Scaffold Manufacturing

Tubular matrices were fabricated using a NANON 01-B electrospinning setup (MECC Inc., Fukudo Ogori-shi, Japan) with a standard clip spinneret and an 8 mm rod rotary collector under the established setup mode, chosen on the base of our previous experience [17]. An amount of 1 mL of working solution was delivered through a 27 G blunt-tip needle at a constant feed rate of 0.5 mL/h. Nanofibers were generated at 16 kV at a collector rotation speed of 300 rpm, spinneret speed of 150 rpm and tip-to collector distance of 15 cm.

### 3.4. Film Fabrication

Film samples were made through pouring 1 g of working solution on a standard glass microscopic slide (76 × 26 mm); the solution was evenly spread over the slide surface through gentle rocking by hand, and then films were left overnight to dry at room temperature. The mass of the applied solution was controlled with analytical-grade weights (Shimadzu Corporation, Kyoto, Japan).

### 3.5. Mechanical Properties Evaluation

For mechanical tests, rectangular fragments 15 × 10 mm in size (*n* = 10 per group) were cut out longitudinally or circumferentially from the obtained tubular electrospun scaffolds. The “dog-bone”-shaped samples were cut out of the films (*n* = 10 per group) with a cutting stamp (overall length of 17.5 mm, overall width of 10 mm, narrow section length of 6 mm, narrow section width of 5 mm). Before tensile tests, the sample thickness (mm) was measured at three points for all samples, and then the average value was calculated. For the “dog-bone” samples, all three points were selected in the narrow section. Measurements were performed with a digital caliper (Mitutoyo, Kawasaki, Japan). Then tested samples were placed in the grips of an ESM 303 L tester (Mark-10 Corporation, Copiague, NY, USA) with a computer-linked force gauge (0–100 N) and stretched until failure at an extension rate of 10 mm/min. All samples were tested dry, without any preconditioning.

Strength was evaluated as failure stress (σ, MPa) (Equation (1)):

(1) $$\sigma =\frac{F\;max}{S}$$

Failure strain (*ε*, %) was calculated using (Equation (2)):

(2) $$\varepsilon =\frac{L\;max-Lo}{Lo}\times 100\%$$

where *ε* is the failure strain, *L_o_* is the initial sample length (mm) equal to the distance between the grips and *L max* is the maximal deformation (mm).

### 3.6. Sample Hardness Measurements

For sample hardness measurements, each working solution was poured in a cylindrical mold of 2 cm in diameter and dried in a fume hood at constant room temperature to obtain samples with a thickness of 6 mm. The measurements were carried out with a Shore D scale durometer (Vostok-7, Moscow, Russia); ten points of measurement were chosen for every sample. The results are represented as mean ± standard deviation.

### 3.7. Brillouin Scattering Spectroscopy

Brillouin spectroscopy is an optical method that measures light inelastically scattered by acoustic phonons. This method allows evaluating the sound velocity in a material from the spectral position of the Brillouin peaks. In the case of materials with a relaxational response slower than a nanosecond, as in the present study, the method provides information on the elastic response in the infinite-frequency limit. Here, we applied the Brillouin spectroscopy method to determine the longitudinal elastic modulus of PCL and PCHC polymers as a function of SWCNT concentration.

The Brillouin experiment was realized in the back-scattering configuration using a 532.1 nm solid-state laser (Spectra Physics, Milpitas, CA, USA) and a 3 + 3 pass tandem interferometer (JRS Scientific Instruments, Zurich, Switzerland). The focusing and collecting lens had a focal length of 150 mm. The typical laser power was a few mW, and control measurements were taken at several-times-lower power to ensure that the effects of laser heating were negligible. The free spectral range was 25 GHz, and the finesse was estimated to be 100. The Brillouin spectra from four different spatial points were measured for each sample.

### 3.8. Scanning Electron Microscopy (SEM)

SEM imaging of matrices was performed using a SU1000 FlexSEM II scanning electron microscope (Hitachi, Tokyo, Japan). Cut samples measuring 50 mm × 50 mm were straightened and fixed appropriately on a specimen tray with conductive tape. Then, samples were sputtered with carbon alloy via a GVC-3000 Thermal Evaporation Carbon Coater (KYKY Technology Co., Beijing, China). Sample observation was performed using a secondary electron detector at an electron high tension of 20 keV. Ten observation fields were selected for every specimen and were examined at 100×, 250×, 450×, 700×, 800× and 1000× magnification. The fiber sizes were measured using a FlexSEM1000 operating program.

### 3.9. Atomic Force Microscopy

An Atomic Force Microscope NTEGRA II (NT-MDT Spectrum Instruments, Moscow, Russia) was used to visualize carbon nanotubes. Each sample was analyzed under semi-contact mode using a silicone ultrasharp probe HA_FM A (NT-MDT Spectrum Instruments, Moscow, Russia). Ten AFM fields of 50 µm × 50 µm and 5 µm × 5 µm were analyzed for each sample at the scan rate of 0.6 Hz. Image analysis software Nova-Px v. 3.5.0 rev. 20351 (NT-MDT Spectrum Instruments, Moscow, Russia) was used to obtain topographic images.

### 3.10. Statistical Analysis

Quantitative data were processed using Dell Statistica 13.0 (Dell Software Inc., Aliso Viejo, CA, USA). Results are presented as mean (M) ± standard deviation (SD). The Mann–Whitney (M-W) U-test was used to compare two groups. The significance level was set to *p* < 0.05 (Tables S1–S8).

## 4. Conclusions

Based on the obtained results, we cannot recommend the studied SWCNT suspension for the fabrication of tissue scaffolds via electrospinning. However, SWCNTs improve the mechanical behavior of polymer poured films, which can be processed using the molding method of scaffold production. We assume that this effect is based on the architecture of the percolative network of nanotubes exposed to different conditions depending on the processing method.

It is noteworthy that even small amounts of SWCNT suspension added to a polymer solution can have a noticeable effect on its physical properties, such as material hardness or stress at break. These effects and their dependence on the SWCNT concentration are not universal for different materials. Some materials, like PCL, seem to have a CNT concentration optimum, which has negative effects on its mechanical properties when it is exceeded.

The perspective of this search is investigating new ways of processing and alternative ways of SWCNT dispergation that would minimize the effect of the suspension additives.

## Figures and Tables

**Table 1:** The position of Brillouin line and the evaluation of the longitudinal elastic modulus of the samples under study. The data are presented as mean ± standard deviation.

Polymer	Parameter	SWCNT Concentration (%)					
		0	0.01	0.02	0.03	0.04	0.05
PCHC	*ν_B_*, GHz	12.26 ± 0.08	12.38 ± 0.03	12.53 ± 0.09	12.54 ± 0.04	12.59 ± 0.03	12.63 ± 0.02
PCL	*ν_B_*, GHz	12.99 ± 0.006	13.00 ± 0.01	13.033 ± 0.01	13.054 ± 0.02	13.066 ± 0.01	13.083 ± 0.02

## Data Availability

The data presented in this study are available as supplementary material for the article.

## Supplementary Materials

The following supporting information can be downloaded at: https://www.mdpi.com/article/10.3390/ijms241311092/s1.
