# Supplementary material for: Influence of Single-Wall Carbon Nanotube Suspension on the Mechanical Properties of Polymeric Films and Electrospun Scaffolds
Source: Int J Mol Sci. 2023 Jul 4;24(13):11092. doi: 10.3390/ijms241311092 (PMC10342290; doi:10.3390/ijms241311092)
Supplement: Supplementary file 1 [file ijms-24-11092-s001.zip › ijms-2404230-supplementary.pdf]

Table S1. Statistical significance of axial SBR data analysis for electrospun PCL tubular matrices.

Test group (SWCNT content, by mass)	Pure PCL	0.01%	0.02%	0.03%	0.04%	0.05%
Pure PCL	—	0.52	0.008	0.003	0.001	0.010
0.01%		—	0.025	0.014	0.008	0.043
0.02%			—	0.298	0.138	0.936
0.03%				—	0.107	0.689
0.04%					—	0.333
0.05%						—

Table S2. Statistical significance of circumferential SBR data analysis for electrospun PCL tubular matrices.

Test group (SWCNT content, by mass)	Pure PCL	0.01%	0.02%	0.03%	0.04%	0.05%
Pure PCL	—	0.525	0.303	0.525	0.303	0.019
0.01%		—	0.109	0.014	0.008	0.014
0.02%			—	0.378	0.012	0.066
0.03%				—	0.081	0.575
0.04%					—	0.747
0.05%						—

Table S3. Statistical significance of axial strain data analysis for electrospun PCL tubular matrices.

Test group (SWCNT content, by mass)	Pure PCL	0.01%	0.02%	0.03%	0.04%	0.05%
Pure PCL	—	0.306	0.212	0.004	0.002	0.026
0.01%		—	0.100	0.027	0.003	0.027
0.02%			—	0.005	0.008	0.128
0.03%				—	1.000	0.229
0.04%					—	0.100
0.05%						—

Table S4. Statistical significance of circumferential strain data analysis for electrospun PCL tubular matrices.

Test group (SWCNT content, by mass)	Pure PCL	0.01%	0.02%	0.03%	0.04%	0.05%
Pure PCL	—	0.088	0.034	0.044	0.022	0.026
0.01%		—	0.008	0.003	0.002	0.003
0.02%			—	0.093	0.074	0.065
0.03%				—	0.617	0.721
0.04%					—	0.830
0.05%						—

Table 5. Statistical significance of SBR data analysis for PCL film samples.

Test group (SWCNT content, by mass)	Pure PCL	0.01%	0.02%	0.03%	0.04%	0.05%
Pure PCL	—	0.012	0.747	0.22	0.272	0.045
0.01%		—	0.137	0.003	0.003	0.002
0.02%			—	0.077	0.019	0.028
0.03%				—	0.467	0.35
0.04%					—	1
0.05%						—

Table 6. Statistical significance of strain data analysis for PCL film samples.

Test group (SWCNT content, by mass)	Pure PCL	0.01%	0.02%	0.03%	0.04%	0.05%
Pure PCL	—	0.22	0.786	0.008	0.097	0.006
0.01%		—	0.307	0.0003	0.0001	0.0004
0.02%			—	0.003	0.018	0.0006
0.03%				—	0.239	0.098
0.04%					—	0.028
0.05%						—

Table 7. Statistical significance of SBR data analysis for PCHC film samples.

Test group (SWCNT content, by mass)	Pure PCHC	0.01%	0.02%	0.03%	0.04%	0.05%
Pure PCHC	—	0.0001	0.836	0.127	0.002	0.0004
0.01%		—	0.0002	0.0002	0.005	0.039
0.02%			—	0.483	0.012	0.0003
0.03%				—	0.012	0.0003
0.04%					—	0.173
0.05%						—

Table 8. Statistical significance of strain data analysis for PCHC film samples.

Test group (SWCNT content, by mass)	Pure PCHC	0.01%	0.02%	0.03%	0.04%	0.05%
Pure PCHC	—	0.26	0.836	0.201	0.591	0.433
0.01%		—	0.365	0.003	0.799	0.158
0.02%			—	0.307	0.169	0.86
0.03%				—	0.018	0.597
0.04%					—	0.149
0.05%						—

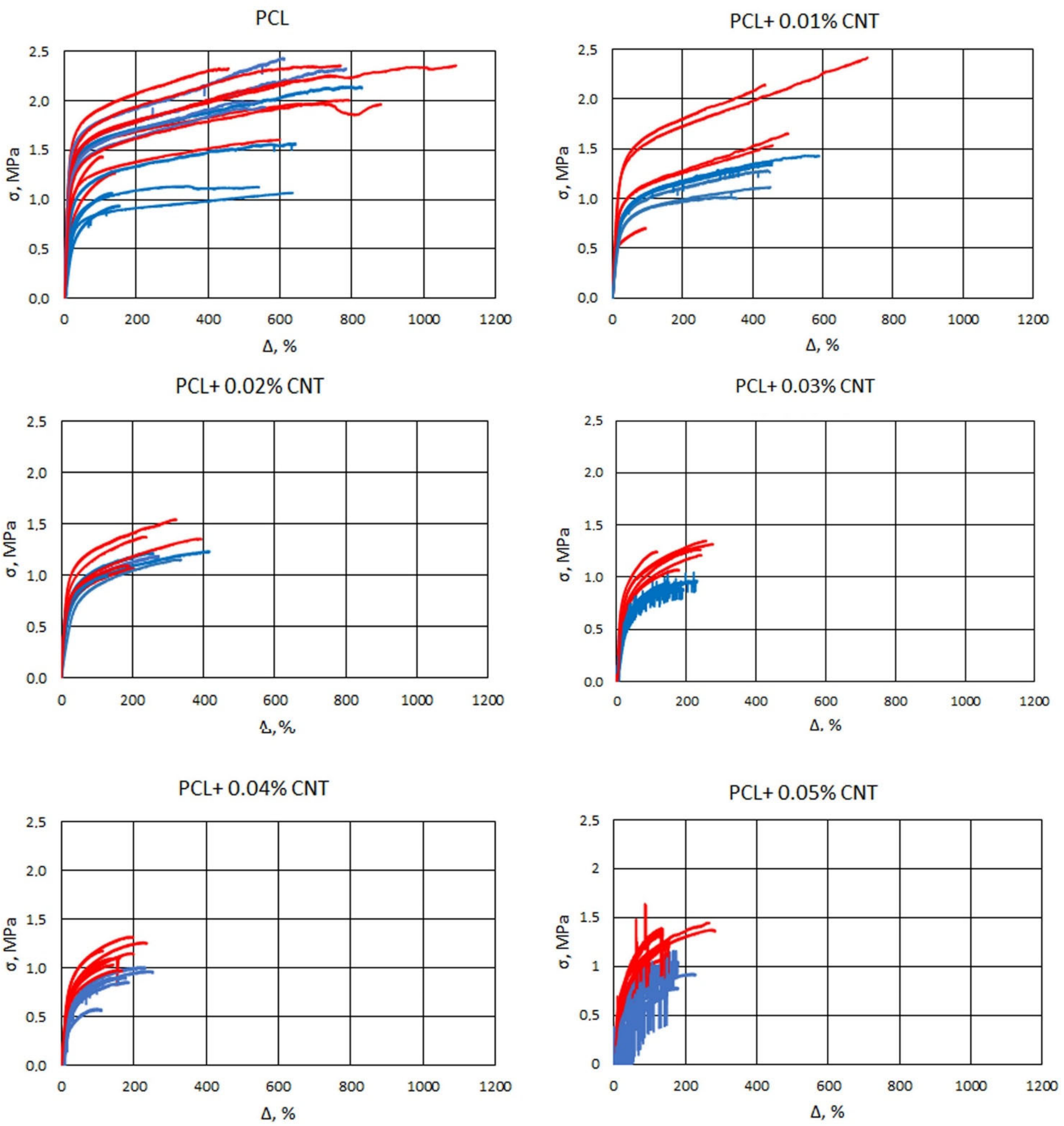

Supplementary figure 1. Strain/stress curves of tested electrospun scaffolds, by SWCNT concentration. Red lines indicate plotting in the axial direction; blue curves correspond to circumferential stretching.

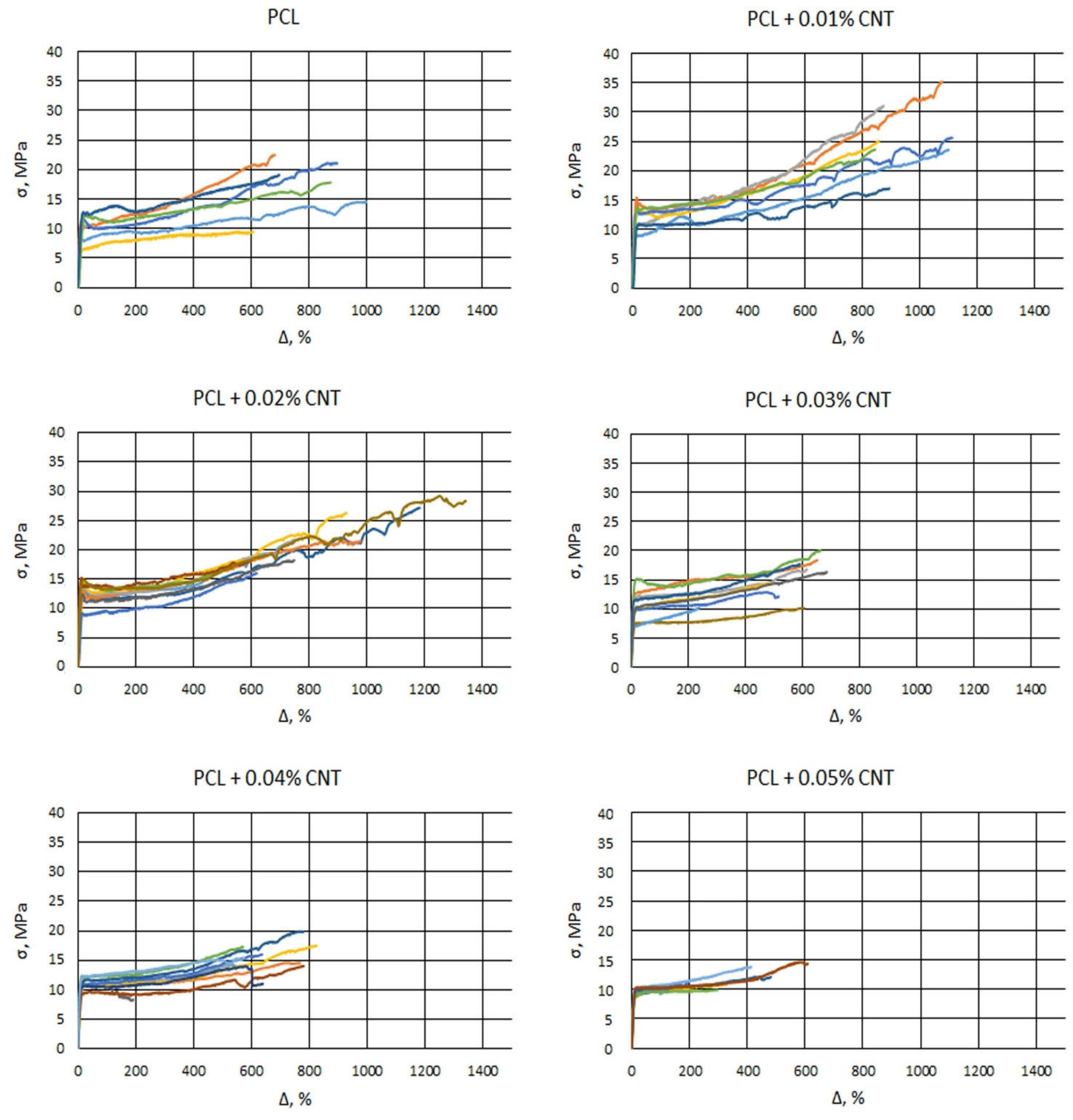

Supplementary figure 2. Strain/stress curves of tested PCL films; different colors indicate different samples.

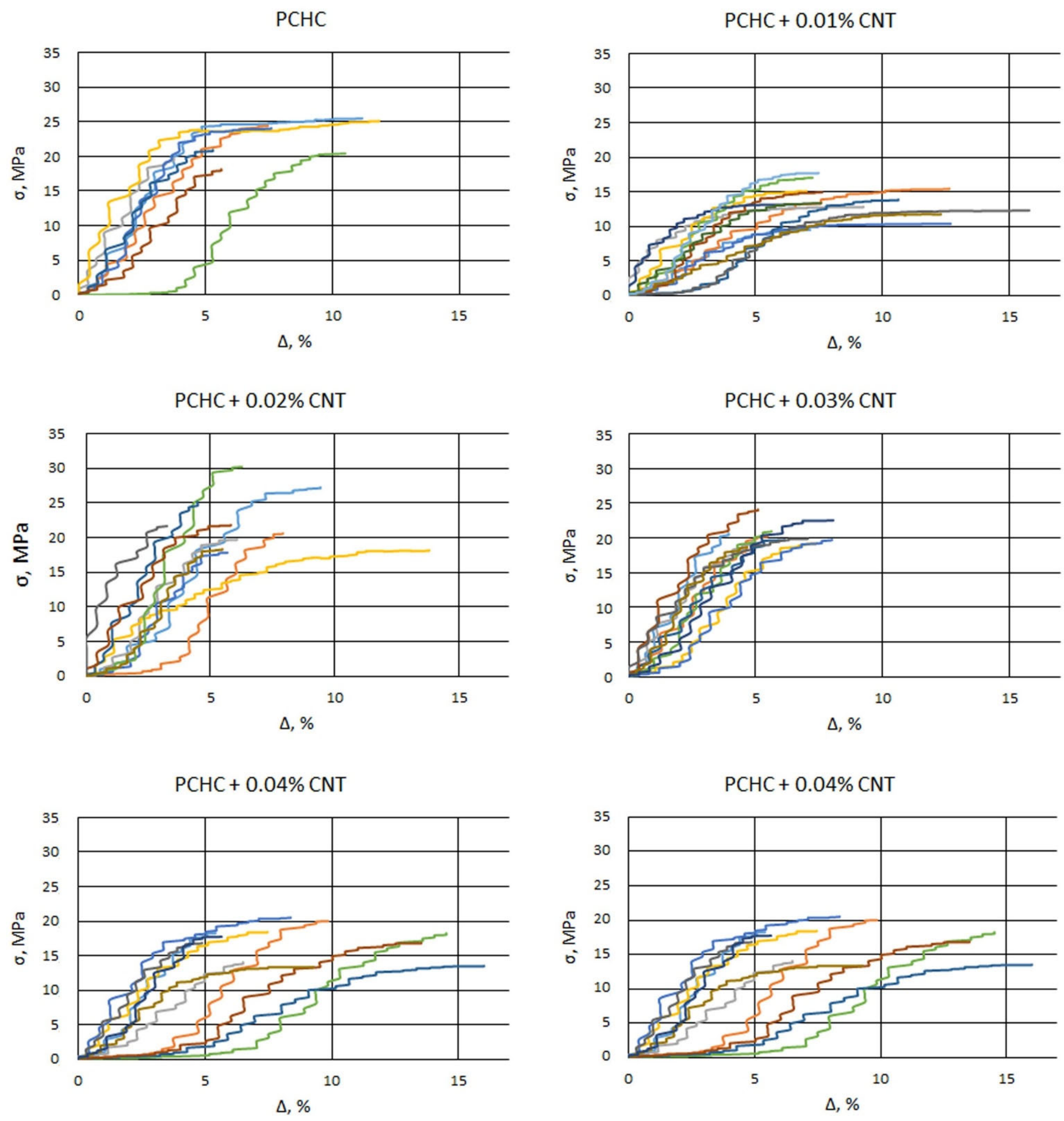

Supplementary figure 3. Strain/stress curves of tested PCHC films; different colors indicate different samples.
